# Comprehensive Insights into Sugar Transporters of *Candidozyma auris* and Their Roles in Antifungal Resistance

**DOI:** 10.3390/jof12020094

**Published:** 2026-01-30

**Authors:** Praveen Kumar, Mohit Kumar, Amandeep Saini, Sheikh Owais Mohamad, Basharat Ali, Brooke D. Esquivel, Atanu Banerjee, Theodore C. White, Naseem A. Gaur, Abdul Haseeb Shah, Amresh Prakash, Rajendra Prasad

**Affiliations:** 1 Amity Institute of Biotechnology, Amity University Gurgaon, Manesar 122413, India; pkumar.kas@gmail.com (P.K.); mohitplawat007@gmail.com (M.K.);; 2 Department of Bioresources, School of Biological Science, University of Kashmir, Srinagar 190006, Indiaabdulhaseeb@kashmiruniversity.ac.in (A.H.S.); 3 School of Life Sciences, Jawaharlal Nehru University, New Delhi 110067, India; 4 School of Science and Engineering, University of Missouri Kansas City, Kansas City, MO 64110, USA; 5 Yeast Biofuel Group, International Centre for Genetic Engineering and Biotechnology, New Delhi 110067, India; naseemgaur@gmail.com

**Keywords:** *Candidozyma auris*, HGT, fluconazole, molecular docking, molecular dynamic simulation

## Abstract

In *Candida* species, including *Candidozyma auris* (formerly *Candida auris*), overexpression of efflux pumps is a well-established mechanism of antifungal resistance. However, accumulating evidence indicates that impaired drug import may also significantly contribute to reduced antifungal susceptibility. Sugar importers, historically viewed solely as hexose transporters (HGTs), are now emerging as potential indirect modulators of antifungal uptake. Here, we performed a comprehensive inventory and functional analysis of the HGT family in *C. auris* to assess its contribution to antifungal import. Phylogenetic analyses revealed that *C. auris* HGTs are more closely related to those of *Candida albicans* (*C. albicans*) than *Saccharomyces cerevisiae* (*S. cerevisiae*). All HGT genes showed basal expression, with several significantly downregulated upon fluconazole (FLC) exposure. To establish functional relevance, we generated a mini-library of HGT deletion mutants. Notably, the Δ*hgt13* strain exhibited markedly increased FLC resistance, concomitant with reduced intracellular FLC accumulation and decreased membrane permeability. Consistently, molecular docking and molecular dynamics simulations demonstrated strong and stable interactions between FLC and Hgt13p. Together, these findings implicate Hgt13p as a key determinant of FLC import and membrane permeability, revealing reduced FLC import could also contribute to antifungal resistance in *C. auris*.

## 1. Introduction

Human fungal infections range from common superficial conditions to life-threatening invasive diseases caused by pathogens like *Candida albicans*, *Aspergillus fumigatus*, *Cryptococcus neoformans*, and the emerging multidrug-resistant *C. auris* [1,2]. These infections are a major threat to immunocompromised individuals, including those with HIV/AIDS, undergoing chemotherapy, or in intensive care units [3]. The rising incidence of antifungal resistance driven by overuse of antifungals in medicine and agriculture has severely limited treatment options. Resistance to all major antifungal classes, including azoles, echinocandins, polyenes, and flucytosine, has complicated management and increased mortality [4]. The emergence of pan-resistant strains of *C. auris* further underscores the urgent need for novel antifungal agents, improved diagnostics, and global stewardship strategies [5].

Antifungal resistance arises through multiple mechanisms that enable fungi to survive otherwise lethal drug exposure. Common strategies include target site alterations, mutations in target genes encoding drug-binding enzymes such as *ERG*11 in azole resistance or *FKS1*/*FKS2* in echinocandin resistance, and reducing drug affinity [4,6]. Overexpression of efflux pumps, particularly ATP-binding cassette (ABC) and major facilitator superfamily (MFS) transporters, actively expels drugs from the cell [7,8,9,10]. Alterations in membrane sterol composition influence the binding of polyenes such as amphotericin B (AMB) and consequently affect their efficacy [10,11,12,13]. Metabolic bypass pathways can circumvent blocked steps in biosynthetic routes, as seen in flucytosine resistance [14]. Biofilm formation further protects fungal cells through physical barriers and a tolerant subpopulation of persister cells [15]. Additionally, stress response pathways and epigenetic changes can transiently enhance tolerance, contributing to persistent fungal infections even in the absence of classical genetic resistance [16,17].

Permeability constraints play a critical role in antifungal resistance by limiting the intracellular accumulation of drugs through both reduced import and enhanced export [18,19]. Exporters play a central role in azole resistance by actively removing drugs from fungal cells, thereby lowering their intracellular concentration below therapeutic levels. The most prominent are ABC transporters, such as *CDR1* and *CDR2* in *C. albicans* and *CDR1* in *C. auris*, which use ATP hydrolysis to efflux a broad range of azoles [20,21]. MFS transporters, notably *MDR1* in *C. albicans*, also contribute to resistance by using the proton motive force to expel drugs [22]. Overexpression of these efflux pumps is often triggered by mutations or hyper activation of transcriptional regulators such as *TAC1*, which regulates *CDR1* in *C. auris* [23], and *MRR1* regulates both *MDR1* and *CDR1* in other *Candida* species [22,24]. This pump-mediated export not only confers resistance to multiple azoles but can also lead to cross-resistance to other structurally unrelated antifungals, making efflux systems a key and clinically significant barrier to effective azole therapy.

Cellular permeability constraints imposed by the overexpression of a battery of efflux proteins (exporters) remain one of the prominent mechanisms of drug resistance employed by most *Candida* species, *C. auris* included [20]. However, more recent evidence suggests reduced drug uptake as another mechanism of drug resistance. Azole antifungals, such as FLC, generally enter fungal cells by facilitated diffusion rather than through dedicated high-affinity importers [25,26]. Their uptake is therefore strongly influenced by the physicochemical properties of the cell membrane. Recent studies have pointed out the existence of transporter proteins mediating entry of azoles under specific condition; however, no conserved, essential azole importer has been identified in pathogenic fungi [25]. Consequently, resistance related to import is largely indirect, arising from membrane remodelling that diminishes facilitated influx, often acting in concert with overexpression of efflux pumps to further reduce effective intracellular drug levels. Although a drug importer remains elusive, compelling evidence does point to the role of drug importer-mediated antifungal resistance [26,27].

Sugar importers, while primarily responsible for transporting monosaccharides and disaccharides to fuel fungal metabolism, can also contribute to antifungal drug resistance through more indirect but increasingly recognized mechanisms [28,29]. In *Candida* and other yeasts, certain hexose transporters and sugar–proton symporters influence the proton motive force and overall membrane potential, which in turn modulates the activity of proton-driven efflux pumps, particularly those of the MFS [30,31]. Sugar transport systems are also linked to nutrient-sensing pathways that regulate stress responses and transporter expression; in nutrient-replete conditions, these pathways can enhance efflux pump production, indirectly promoting azole resistance [32]. Additionally, shifts in carbon source utilization can remodel membrane lipid composition and cell wall architecture, further affecting drug permeability and susceptibility. Recently, *HXT4/6/7* have been linked to azole import in *Nakaseomyces glabratus* (formerly called *Candida glabrata*), wherein mutations in these genes altered their import capacity, resulting in the development of FLC resistance [28]. In another context, the *HGT13* in *S. cerevisiae* was reported to mediate efflux of the antifungal drug miltefosine, functioning as an efflux pump and thereby reducing intracellular drug accumulation [33]. Similarly, deletion of *HXT11* and *HXT9* in *S. cerevisiae* conferred resistance to cycloheximide, sulfometuron methyl, and 4-nitroquinoline-N-oxide (4-NQO), further supporting the role of HGTs in drug import [34]. Additionally, several multi-omics studies of *C. auris* reported upregulation of several HGT genes, implying their role in FLC susceptibility [9,35].

To determine whether the sugar transporters in the WHO-designated critical-priority fungal pathogen *C. auris* modulate drug import and FLC susceptibility, we conducted a comprehensive analysis of the HGT gene family, coupled with species-specific and comparative phylogenetic studies across related fungi. Antifungal susceptibility testing (AFST) and functional assays revealed that all HGT genes exhibit basal expression, with most showing downregulation under FLC-induced stress. Among the tested HGT genes, Δ*hgt13* cells appeared to inhibit facilitate of FLC import, suggesting a possible role in resistance. Molecular docking and simulation studies of Hgt13p demonstrated a high affinity for FLC binding, involving conserved critical residues, thereby supporting its function as a potential drug importer.

## 2. Materials and Methods

### 2.1. Strains and Media

We used the *C. auris* clade II isolate B11220 (CBS10913T) as the wild type and constructed mutants in this strain. All media components and reagents were of molecular-grade quality and procured from standard commercial suppliers. Growth media included yeast extract, peptone, and dextrose (YPD) (HiMedia, India), along with chemicals such as dimethyl sulfoxide (DMSO) (Sigma, MO, USA) and FLC (Merck, MO, USA). The PCR purification kit and genomic cDNA kit were obtained from Thermo Fisher Scientific Baltics, UAB.

### 2.2. Phylogenetic Analysis of Hgt13p with HGTs

The predicted HGT protein sequences of the *C. auris* were retrieved from the *Candida* genome database (CGD), although with the *C. auris* sequence, we also retrieved all HGT protein sequences of *C. albicans* and *N. glabratus* from the CGD database, along with the HGT protein of the *S. cerevisiae* from the Saccharomyces Genome Database (SGD). To confirm the identity of the 15 putative proteins, we performed BLASTp analysis. To achieve this, each *C. auris* HGT protein sequence was BLAST (BLAST+2.17.0) and analyzed against the *C. albicans* and *S. cerevisiae* genomes, and sequences were validated as HGTs based on their percent identity and statistical significance (*p*-value) (Supplementary Table S1). For the phylogeny construction of *C. auris*, protein sequences of 15 sugar transporters underwent multiple sequence alignment (MSA) of the MUSCLE algorithm using MEGA version 11. The MSA output was directly fed into phylogenetic analysis of the 15 *C. auris* transporters using the Maximum Likelihood method with 1000 bootstrap replicates, providing statistical confidence by resampling alignments to test branch robustness. The same procedure was applied to construct a multispecies phylogeny of all sugar transporters of *C. albicans*, *N. glabratus*, *S. cerevisiae*, and *C. auris* using MEGA version 11 followed by Maximum Likelihood phylogeny with identical bootstrapping. This enables cross-species evolutionary inference, highlighting conserved motifs and divergences in transporter families.

### 2.3. RNA Extraction

RNA was isolated using the hot phenol method. Yeast cells were harvested after cultivation, centrifuged at 4000 rpm for 5 min, and washed twice with ice-cold DEPC-treated (Sigma, MO, USA) water. The pellet was resuspended in 400 µL TES buffer, mixed with 400 µL acid phenol (HiMedia, India), vortexed, and incubated at 65 °C for 60 min with intermittent mixing. After centrifugation at 4 °C for 5 min, the aqueous phase was extracted twice with acid phenol and twice with chloroform (Honeywell, Germany). RNA was precipitated with 2–2.5 volumes of 100% ethanol (Merck, USA) and 10% 3M NaOAc (pH 5.2) for 1 h on ice, centrifuged at 4 °C for 15 min, washed with 70% ethanol, air-dried, and dissolved in RNase-free water. RNA concentration was determined using NanoDrop (Thermo Fisher scientific, Waltham, MA, USA), and stored at −80 °C for further use.

### 2.4. Quantitative Real Time-PCR and Analysis

#### 2.4.1. DNase I Treatment

Residual DNA contamination was removed using Thermo Fisher Scientific DNase I. A 10 µL reaction containing 5 µg RNA, 1 U DNase I, and 1X DNase I buffer was incubated at 37 °C for 60 min, followed by enzyme inactivation with 0.5 µL of 25 mM EDTA at 65 °C for 10 min. RNA integrity and DNA removal were verified by PCR amplification of the *CauTDH3* gene, with no product indicating successful digestion.

#### 2.4.2. cDNA Synthesis

First-strand cDNA was synthesized using the RevertAid H Minus First Strand cDNA Synthesis Kit (Thermo Fisher Scientific Baltics, UAB). A 12 µL mixture containing 1 µg DNase-treated RNA, 50 µM oligo(dT), and 50 µM random primers was incubated at 65 °C for 5 min. An 8 µL cDNA synthesis mix (including Riboloc, reverse transcriptase, 5X buffer, and dNTPs) was added, followed by incubation at 42 °C for 60 min and 75 °C for 5 min. cDNA quality was confirmed by successful *CauTDH3* amplification.

### 2.5. Quantitative Real-Time PCR and Analysis

Total RNA was quantified using a NanoDrop 2000 spectrophotometer. cDNA was synthesized from 1 µg RNA using the RevertAid H Minus First Strand cDNA Synthesis Kit (Thermo Fisher Scientific, Baltics, UAB). A 12 µL mixture containing 1 µg DNase-treated RNA, 50 µM oligo(dT), and 50 µM random hexamer (Cat. No. K2562). Quantitative gene expression was analyzed with the CFX96 real-time PCR system (Bio-Rad, Hercules, CA, USA) using gene-specific primers (Supplementary Table S2) and iTaq Universal SYBR Green Supermix (Bio-Rad; Cat. No. 172-1544, Hercules, CA, USA). Expression levels were normalized to the *CauTDH3* housekeeping gene, and relative quantification was performed using the 2^−ΔCT^ method. All reactions were conducted in technical triplicates and biological duplicates.

### 2.6. Construction of HGT Gene Deletion

*C. auris* ORFs were disrupted by homologous recombination using a fusion PCR-based cassette containing the *NAT1* gene, which encodes nourseothricin acetyltransferase and confers resistance to nourseothricin. The 5′ and 3′ UTR flanking regions of the target gene were PCR-amplified by using gene-specific primers from wild-type genomic DNA, while two *NAT1* fragments containing FRT sites were amplified using the *NAT1* cassette-specific primers from plasmid pRK625. Each UTR fragment was fused to one half of the *NAT1* cassette, with an overlapping region of ~300–350 bp between the two *NAT1* halves. All oligonucleotides (G-Bioscience, USA) used in the construction of each gene are given in Supplementary Table S3.

For the transformation of *C. auris*, the fused PCR constructs were co-transformed into the wild-type strain using a modified lithium acetate/PEG method [36]. *C. auris* was cultured overnight in YPD at 30 °C at 200 rpm and then subcultured to an OD_600_ of 0.1 and grown to 0.6–0.8. After that, the cells were harvested at 4000 rpm and washed thrice with sterile water, and cell pellets were kept on ice and resuspended in 50 µL of 100 mM lithium acetate. A transformation mix containing 50% PEG (Sigma, USA), 1M lithium acetate (Sigma, USA), denatured ssDNA (10 mg/mL) (Sigma, USA), and 500–1000 ng transforming DNA (final volume 360 µL) was added and incubated at 30 °C for 45 min. After adding 43 µL DMSO, cells were heat-shocked at 42 °C for 15 min and incubated on ice for 5 min. cells were centrifuged at 4000 rpm for 5 min, and cell pellets were resuspended and incubated in 1mL YPD broth for 1.5–2 h. After centrifugation, cells were then plated on YPD agar containing 200 µg/mL nourseothricin (GoldBio MO, USA). Plates were incubated at 30 °C for 24–48 h until positive colonies appeared. Successful gene disruption was confirmed by PCR using specific primers flanking the recombination sites (Supplementary Table S3).

### 2.7. Minimum Inhibitory Concentration (MIC)

MIC tests were carried according to Clinical and Laboratory Standards Institute (CLSI) protocols with some modifications [37]. YPD media was used to culture *C. auris* cells at 30 °C overnight, with an OD_600_ nm of 0.1; the cells were diluted in 0.9% saline solution. After that, the cells were diluted 100 times in YPD media. Equal amounts of media and various FLC (Merck, MO, USA) concentrations were present in the wells of round-bottomed, 96-well microtiter plates before the diluted cell suspension was added. For 48 h, the plates were incubated at 30 °C. Using microplate reader (Bio-Rad iMark, Hercules, CA, USA) the optical density at OD_600_ nm was used to evaluate the MIC test endpoint, which was well-defined as the lowest antifungal drug concentration that produced 50% inhibition of growth (MIC_50_) in comparison to the growth of an FLC-free positive control.

### 2.8. ^3^H-FLC Accumulation Assay

The ^3^H-FLC accumulation assay followed established guidelines [38]. Cells were grown for 16 h, washed three times with YNB, and starved of glucose for 3 hrs. Samples were then treated in technical triplicate with ^3^H-FLC in YNB ± 2% glucose, shaken, and incubated for 24 h at room temperature. Post-incubation, OD_600_ was measured for each sample. Samples were mixed with stop solution (YNB + 20 mM FLC), washed once more with YNB, and vacuum-filtered over glass microfiber filters. Filters were rinsed and dipped in scintillation fluid, and radioactivity was measured as counts per minute (CPM). Accumulation values were normalized to CPM per 10^8^ cells using the pre-filtration OD_600_. For ^3^H-FLC efflux assays, preloaded samples were washed and resuspended in fresh YNB ± 2% glucose, and intracellular ^3^H-FLC levels were quantified at specified time intervals.

### 2.9. Membrane Permeability Assay Using NPN-Based Assay

N-phenyl-1-naphthylamine (NPN) (Sigma, MO, USA), a hydrophobic dye, was used to assess membrane permeability with modification. NPN exhibits high fluorescence upon binding to phospholipids but low fluorescence in aqueous solution [39]. Wild-type and Δ*hgt13 Candida* strains were cultured overnight at 30 °C at 200 rpm, washed twice with milli-Q water, and resuspended to 1 × 10^5^ cells/mL. Cells were incubated with 10 µM NPN for 20 min at 37 °C, and fluorescence was measured every 5 min until saturation. Subsequently, NPN-labelled cells were treated with 0.01% SDS (sub-MIC), and fluorescence intensity was recorded every 5 min using a spectrophotometer (λ_ex_ = 350 nm, λ_em_ = 420 nm).

### 2.10. Glucose Accumulation Assay

To assess the impact of Δ*hgt13* on glucose accumulation; assays were carried out using both the Δ*hgt13* mutant and wild-type *C. auris* cells. Overnight cultures grown in YPD at 30 °C with shaking at 200 rpm were harvested by centrifugation at 4000 rpm for 5 min. Secondary culture (OD_600_ = 0.1) was set up in 10 mL YPD containing 2% glucose. The cells were harvested and the resulting cell pellet was washed with sterilized 0.9% saline. The cell pellet used to determine the glucose content inside the cells was resuspended in 300 µL sterile water, boiled for 10 min at 100 °C, and centrifuged, and the supernatant was collected. The intracellular glucose content in the samples was determined using a glucose assay kit (Merck, USA) using the standard glucose control and following the manufacturer’s recommended protocol.

### 2.11. Homology Modelling and Docking

The three-dimensional structure of Hgt13p was generated using the AlphaFold3 server. The crystal structure of the *E. coli* XylE transporter (PDB ID: 4GZB) with D-glucose served as the reference for the structural analysis of glucose binding with Hgt13p [40]. The SwissDock platform was used for the molecular docking of ligand D-glucose with XylE (Figure 3a), using a grid box of size 26 Å × 26 Å × 26 Å, with the sampling exhaustivity 51 [41,42]. The same approach was applied for docking of ligands, D-glucose, and FLC with Hgt13p (Figure 3b,c), with the same grid box size 26 Å × 26 Å × 26 Å as used for the XylE protein, was centred over the interactive residues of the corresponding XylE–D-glucose complex, conserved within Hgt13p. Out of the 20 best poses, the structure with the lowest RMSD and high binding affinity was selected for molecular interaction analysis, using PyMOL (Version 2.5.2).

### 2.12. MD Simulation Setup

Molecular dynamics (MD) simulations of Hgt13p and its complexes with D-glucose and FLC were performed using GROMACS 2023.1 [43] with the CHARMM36 force field [44]. The protein and docked complexes were solvated in a cubic box using the TIP3P water model, maintaining a minimum distance of 1.0 nm between the protein surface and the box edge. Ligand parameters were generated using the CHARMM General Force Field (CGenFF) workflow and converted to Gromacs topology (cgenff_charmm2gmx.py) [45]. A symmetric POPC bilayer was constructed containing ~128 lipids (64 per leaflet) using CHARMM-GUI Membrane Builder [46]. The protein–ligand complex was placed approximately centred in the membrane, such that membrane-facing regions of the protein aligned with the bilayer normal (z-axis). Overlapping lipids within 0.12 nm of protein heavy atoms were removed. The counter ions (Na^+^/Cl^−^) were added to neutralize the system. Energy minimization was conducted using the steepest descent algorithm and conjugate gradient until the system reached a convergence threshold of 1000 kJ/mol/nm, respectively. The system was equilibrated under NVT and NPT ensembles for 100 ps each at 300 K and 1 bar using the V-rescale thermostat and Parrinello–Rahman barostat, respectively. The LINCS algorithm was applied to constrain all bond lengths, and long-range electrostatic interactions were treated with the Particle Mesh Ewald (PME) method. Finally, production MD simulations were run for 250 ns with a time step integration of 2fs, and coordinates were saved at an interval of every 10 ps.

### 2.13. Trajectory Analysis

The MD trajectories were analysed using standard GROMACS utilities for root mean square deviation (RMSD), radius of gyration (Rg), and solvent-accessible surface area (SASA) for defining the global structural stability, whereas the free energy landscape (FEL) was constructed using RMSD and Rg as reaction coordinates to identify thermodynamically favourable conformational states [47,48].

### 2.14. MM-GBSA Free Energy Calculation

Binding free energy calculations were performed using the MM-GBSA (Molecular Mechanics/Generalized Born Surface Area) method as implemented in the gmx_MMPBSA package [47,48]. Representative snapshots extracted from the equilibrated 250 ns MD trajectories were used for analysis. The total binding free energy (ΔG_bind_) was estimated as the sum of van der Waals (ΔE_vdW_), electrostatic (ΔE_EEL_), polar solvation (ΔG_GB_), and nonpolar solvation (ΔG_SA_). The energy decomposition plots were used for identifying the spatial contribution of the Hgt13p active site residues in stabilising the ligands, governing the stability of the Hgt13p–ligand complexes.

## 3. Results

### 3.1. Genome-Wide Identification, Homology, and Phylogenetic Analysis of HGT Genes in C. auris

To explore the number of putative HGT genes in *C. auris*, we queried the *Candida* Genome Database (CGD) [http://www.candidagenome.org (10 September 2025)] using the updated genome assembly GCA_002759435.3 of clade I reference strain B8441v3 [49]. The corresponding 15 HGT protein sequences were retrieved in FASTA.

**Figure 1:**
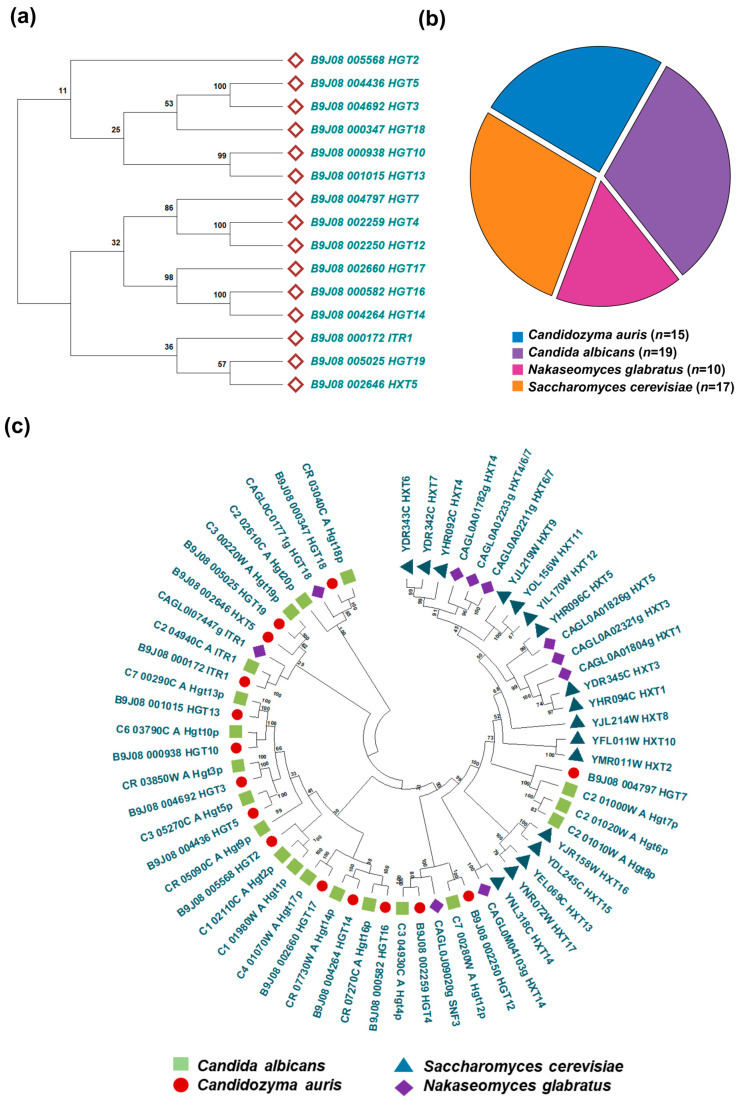
HGT gene repertoire and phylogenetic relationships in *Candida* species. (**a**) Phylogenetic tree of *C. auris* HGT genes constructed using the maximum likelihood method, showing evolutionary clustering patterns and potential functional groupings within the HGT and related transporter families. Bootstrap values from 1000 replicates are indicated at branch nodes. (**b**) Comparative distribution of predicted HGT genes across multiple *Candida* species and *S. cerevisiae*, illustrating species-specific variation in transporter abundance. (**c**) Comprehensive inventory of annotated HGT genes in *Candida* species and *S. cerevisiae*, grouped by transporter family.

To gain deeper insights into the evolutionary relationships of *C. auris* HGTs, we constructed a phylogenetic tree based on the protein sequences of all 15 putative HGTs (Figure 1a). The resulting tree resolved into two major branches, likely indicating potential functional divergence within the *C. auris* HGT family. For comparative analysis, the complete sets of HGT protein sequences from *C. albicans* and *N. glabratus* were also retrieved. In addition, the full repertoire of hexose transporter (HXT) protein sequences from *S. cerevisiae* was retrieved from the Saccharomyces Genome Database (SGD) [https://www.yeastgenome.org (12 September 2025)]. The number of HGT genes varied across species, with *C. albicans* containing 19 genes, *S. cerevisiae* containing 17 genes, *C. auris* containing 15 genes, and *N. glabratus* containing 10 genes (Figure 1b). Phylogenetic analyses were performed to validate transporter identity and assess evolutionary conservation. The comparative maximum likelihood phylogeny revealed that *C. auris* HGTs cluster more closely with orthologs in *C. albicans* than with those from *N. glabratus* and *S. cerevisiae* (Figure 1c). To validate the identity of the 15 predicted *C. auris* HGT genes, we performed BLASTp (protein–protein BLAST) analysis against the protein databases of *C. albicans* and *S. cerevisiae*. The comparative protein homology analysis revealed that the *C. auris* HGT protein shared sequence identities ranging from 50 to 79% with *C. albicans*, whereas the identities with *S. cerevisiae* were comparatively lower (Supplementary Table S1). These results support the evolutionary conservation of HGT genes between *C. auris* and *C. albicans*, while indicating a greater divergence from *S. cerevisiae*.

### 3.2. HGTs Exhibit Conserved Sugar-Transporter Domains with Variable Numbers of Transmembrane Helices

We examined the conserved evolutionary and structural features of the 15 predicted *C. auris* HGTs by analysing their protein sequences using InterProScan, which integrates multiple signature-recognition platforms, including Pfam, PROSITE, SMART, and TIGRFAMs. The analysis detected conserved HGT domains across all HGTs (Supplementary Figure S1a). The size of these conserved domains varied among the transporters, likely reflecting differences in the total length of the respective proteins. Despite this variability, the evolutionary conservation of the domains indicates that these transporters retain the core structural elements essential for substrate recognition and translocation, while also allowing functional diversification.

At the structural level, the HGT genes of *C. auris* are predicted to encode membrane-bound proteins belonging to the major MFS, a group typically characterized by 12 transmembrane (TM) helices arranged into two pseudo-symmetrical bundles, as described for other fungi [50,51]. To further evaluate this feature, we performed AlphaFold3, a deep learning-based algorithm capable of predicting protein structures with near-experimental accuracy [52]. Structural models of the 15 transporters revealed variation in both the number and arrangement of TM helices, with the number ranging from 9 to 12 helices (Supplementary Figure S1b). Notably, the genomic regions corresponding to these TM domains were not entirely conserved across the transporter genes, indicating potential topological diversity within the *C. auris* HGT family. Such variation in the number and arrangement of TM helices may reflect functional diversification among the *C. auris* HGTs. Collectively, these results highlight the evolutionary conservation of HGTs in *C. auris*, while also revealing structural heterogeneity that may underpin specialized functional roles.

### 3.3. HGT Genes Show Distinct Expression Patterns Under Basal and Fluconazole-Induced Conditions

**Figure 2:**
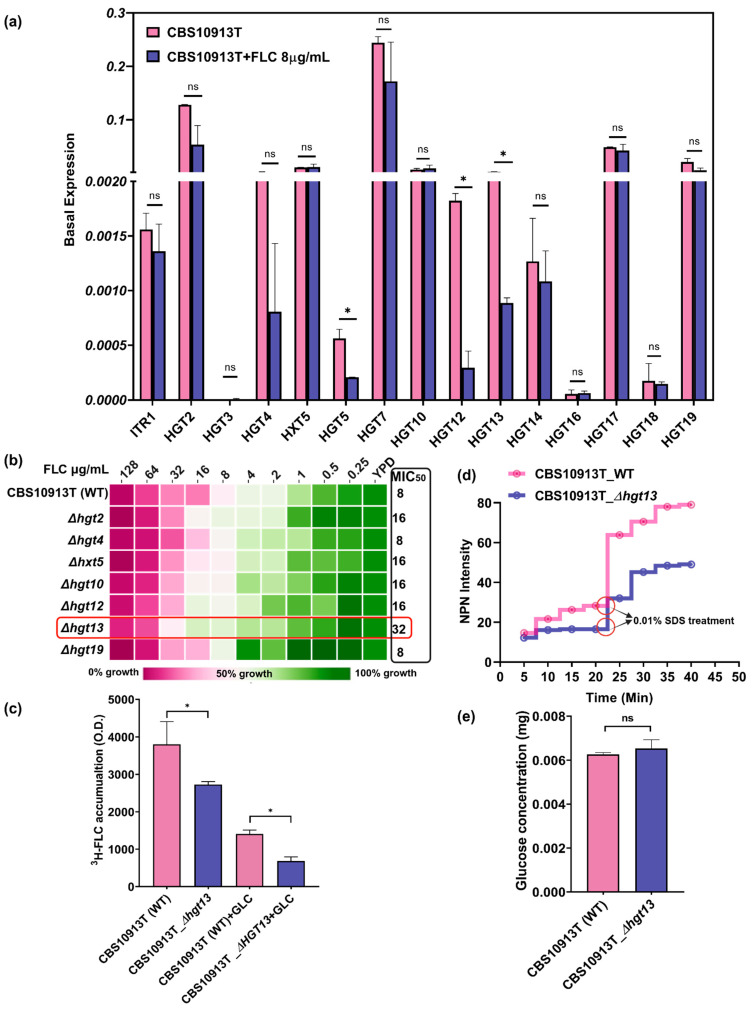
Functional analysis of putative HGT genes in *C. auris*. (**a**) Quantitative real-time PCR (qRT-PCR) analysis of 15 predicted HGT genes in the wild-type azole-susceptible strain CBS10913T. Expression values were calculated using the 2^−ΔCT^ method and normalized to an internal control gene, *CauTDH3*. Data represent the mean ± SD from three independent biological replicates. (**b**) Drug susceptibility using the minimum inhibitory concentration of the Δ*hgt13* with FLC in YPD media shows significant changes marked in the red square box. (**c**) ^3^H-FLC accumulation assay revealed that Δ*hgt13* cells contained significantly less FLC in presence and absence of glucose comparative to the control. Data shows the mean ± SD from three independent biological replicates. (**d**) Membrane permeability assay using the NPN dye shows that the Δ*hgt13* mutant has a more rigid membrane. Data represented as average of three independent experiments. (**e**) The glucose accumulation assay showed no significant difference in glucose uptake between the Δ*hgt13* mutant and the wild-type strain. Data represented as an average of three independent experiments. Significance was calculated using Student’s *t*-test, * *p* < 0.05; ns—not significant.

To investigate the role of HGTs in *C. auris*, we conducted quantitative real-time PCR (qRT-PCR) analysis to evaluate the basal and FLC-induced expression of 15 predicted HGT genes. We observed that several HGT genes, *HGT2*, *HGT5*, *HGT7*, *HGT10*, *HGT17*, and *HGT19*, exhibited higher basal expression levels than the other HGTs. To explore the relationship between HGT gene expression and antifungal resistance, we analysed transcriptional changes upon exposure to FLC. The FLC-exposed cells exhibited downregulation of several HGT genes, including *HGT2*, *HGT4*, *HGT5*, *HGT7*, *HGT12*, *HGT13*, and *HGT14*, compared to the control (Figure 2a). Notably, the expression of several other HGT genes (*HGT3*, *HXT5*, *HGT10*, *HGT16*, *HGT17*, *HGT18*, and *HGT19*) remained unchanged upon FLC treatment. This transcriptional repression may suggest a potential suppression of transporter activity with FLC treatment, which may reflect a broader cellular strategy to minimise drug uptake via facilitated diffusion.

### 3.4. Δhgt13 Exhibits Enhanced FLC Resistance and Reduced Intracellular Drug Accumulation

To investigate the functional role of HGTs, we deleted the downregulated genes (*HGT2*, *HGT4*, *HGT12*, and *HGT13*) and two genes (*HXT5* and *HGT19*) whose expression remained non-responsive to drug exposure in CBS10913T using a fusion PCR-based gene disruption method (Table 1). To examine the role of HGTs in antifungal resistance, we performed antifungal susceptibility testing with HGT mutants. Among the mutants, Δ*hgt13* exhibited a 4-fold increase in MIC_50_ compared to the wild-type strain. At the same time, other HGT deletants did not show any change in FLC resistance (Figure 2b). To further elucidate the role of *HGT13* in FLC susceptibility, we quantified the intracellular accumulation of FLC (^3^H-FLC) in Δ*hgt13* (Figure 2c). In the absence of glucose and thus limited ABC transporter activity, Δ*hgt13* cells exhibited approximately 4-fold less ^3^H-FLC accumulation than the wild type, indicating reduced drug import. Upon glucose addition (Figure 2c, +GLC), both strains showed a similar decline in intracellular ^3^H-FLC compared to the glucose deplete conditions, consistent with the activation of efflux. However, even with efflux transporters active in both strains, the Δ*hgt13* cells still had significantly less intracellular FLC than the wild type, further indicating an efflux-independent mechanism of reduced FLC accumulation in the Δ*hgt13* strain. Collectively, these findings suggest that *HGT13* may facilitate the import of FLC in *C. auris*.

### 3.5. Δhgt13 Elevates Membrane Rigidity Independently of Glucose Uptake

*HGT13* encodes a membrane-localised transporter whose deletion may perturb membrane integrity and diffusion. To check, membrane integrity was assessed using the hydrophobic dye N-phenyl-1-naphthylamine (NPN), which exhibits minimal fluorescence in aqueous environments but becomes strongly fluorescent upon interaction with phospholipids in perturbed membranes [39]. Δ*hgt13* cells showed a consistent reduction in fluorescence over time compared to the WT strain, suggesting reduced membrane accessibility to NPN. Upon SDS treatment, fluorescence further decreased in the mutant compared to the wild type (Figure 2d), pointing to increased membrane rigidity. Notably, no difference in glucose accumulation was observed between Δ*hgt13* and its wild-type strains (Figure 2e).

### 3.6. Molecular Modelling and Docking of Hgt13p

**Figure 3:**
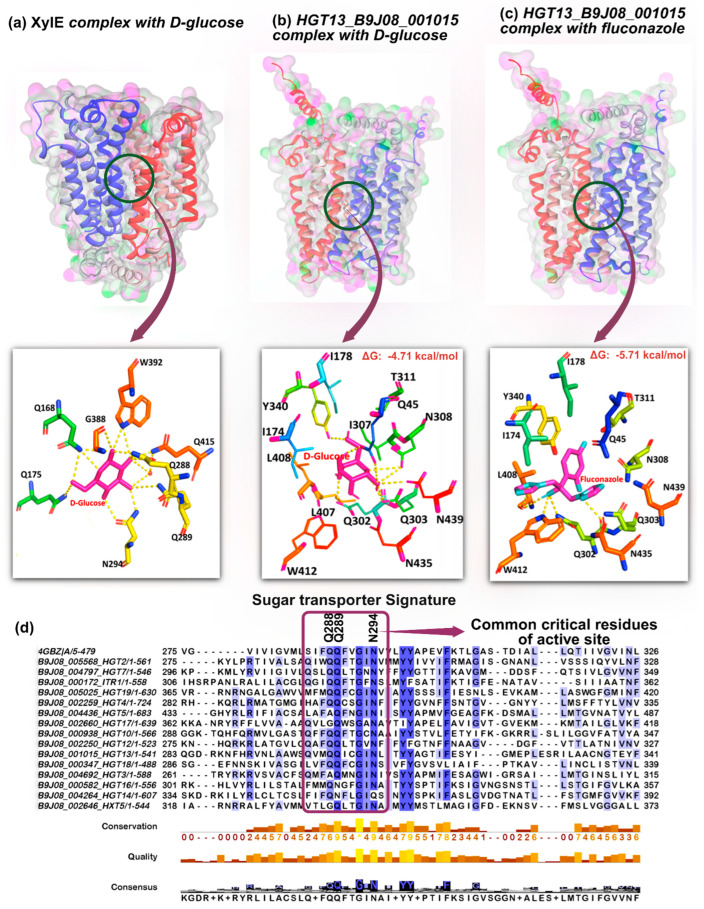
In silico docking analysis of Hgt13p with D-glucose and FLC and comparison with bacterial XylE permease. (**a**) Crystal structure of *Escherichia coli* XylE (PDB ID: 4GBZ) in complex with D-glucose. The substrate-binding pocket representing XylE protein’s interaction with glucose, depicted as magenta lines. Key interacting residues of active site are shown in different-coloured lines, highlighting hydrogen bonds (dashed yellow lines) stabilizing D-glucose. (**b**) Predicted Hgt13p 3D structure generated via AlphaFold3 server, showing the docked complex with D-glucose where magenta lines represent the D-glucose molecule in the binding cavity. Interacting residues are shown in different-coloured lines, with hydrogen bonds indicated by dashed lines. (**c**) Predicted Hgt13p 3D structure generated via AlphaFold3 server, showing the docked complex with FLC where combination of magenta and cyan color lines represent the FLC in the binding cavity. (**d**) Multiple sequence alignment of all 15 *C. auris* HGT proteins with XylE, highlighting conserved substrate-interacting residues. Three critical interacting residues (Gln288, Gln289, and Asn294 from XylE) are conserved from bacteria to yeast and maintained across the HGT family of *C. auris*.

To understand the molecular interaction of D-glucose and FLC with Hgt13p, the three-dimensional structure of the protein was predicted by the AlphaFold3 server [52]. The XylE protein served as a reference to map the structure of Hgt13p, a well-characterized member of the MFS transporter family, enabling the identification of the molecular interactions responsible for binding and transport of D-glucose (Supplementary Figure S2a). The active site residues of the crystalized complex XylE–D-glucose consist of Gln168, Gln175, Gln288, Gln289, Asn294, Gly388, Trp392, Gln415, Ile171, Ile172, Leu297, Tyr298, Gln325, Phe383, Trp387, and Gln419, which are crucial for molecular interactions at the binding pocket (Figure 3a) [40]. However, the crucial interactions of D-glucose with XylE show that the ligand is largely stylized by hydrogen bond interactions with Gln168, Gln175, Gln288, Q289, Asn294, Gly388, and Trp392. To examine the interaction of D-glucose, we additionally performed docking analyses, which yielded consistent results [40]. Docking of D-glucose with the XylE protein revealed that it engages the same active-site region, forming hydrogen-bond interactions specifically with residues Gln175, Gln288, Gln289, Asn294, Asn325, Gly388, and Trp392. Furthermore, to characterise the binding site of Hgt13p, we superimposed its structure with that of the binding site of XylE protein (Supplementary Figure S2b). The structural alignment indicates that the active site of Hgt13p consists of residues that occupy a comparable substrate-binding region, suggesting a conserved architecture underlying ligand recognition (Supplementary Figure S3). The docking of D-glucose with Hgt13p shows hydrogen bond interactions with Gln45, Gln302, Gln303, Tyr340, Asn308, and Asn439 (Figure 3b). Notably, the glucose-binding residues (Gln302, Gln303, and Asn308) of Hgt13p occupy analogous positions to the glucose-interacting residues (Gln288, Gln289, and Asn294) in XylE, suggesting the structural conservation of the key substrate-binding domain of HGT proteins. Furthermore, the docking of FLC with Hgt13p revealed polar interactions with Leu408 and Trp412, in addition to Gln302 and Gln303, which also serve as key interaction sites for D-glucose (Figure 3c). This overlap in binding residues indicates that FLC can access the predicted substrate-binding cavity of Hgt13p; however, FLC is largely stabilised by hydrophobic interactions with the active site residues along with hydrogen bond interactions. This observation is further supported by the multiple sequence alignment of all 15 HGT proteins, along with XylE, which demonstrated conserved substrate-interacting residues across the HGT family in *C. auris* (Figure 3d). Remarkably, three critical XylE residues, Gln288, Gln289, and Asn294, are invariant from bacteria to yeast, underscoring their evolutionary importance in sugar molecule transportation, and these residues observed to be consistent in the HGT family and present within the HGT signature (Figure 3d). 

### 3.7. MD Simulations Indicate a High-Affinity Interaction Between Hgt13p and FLC, Supported by Conserved Active-Site Residues

To further validate the molecular interactions and spatial binding stability of D-glucose and FLC with Hgt13p, we performed MD simulations of the docked complexes embedded within a POPC bilayer membrane under physiologically relevant conditions for a period of 250 ns. The structural stability and conformational dynamics of docked complexes were examined using RMSD, Rg, SASA, and RMSF analyses, while hydrogen-bond profiles defined specific ligand interactions. Ligand stability was assessed via ligand-RMSD, and free energy landscape (FEL) analysis identified energetically favourable conformations. The binding free energy (ΔG_bind_) was further estimated using MM-GBSA calculations, offering a quantitative insight into molecular interactions in energetic terms, defining the binding stability and affinity of ligands with Hgt13p.

#### 3.7.1. Structural Stability of Hgt13p and Docked Complexes

**Figure 4:**
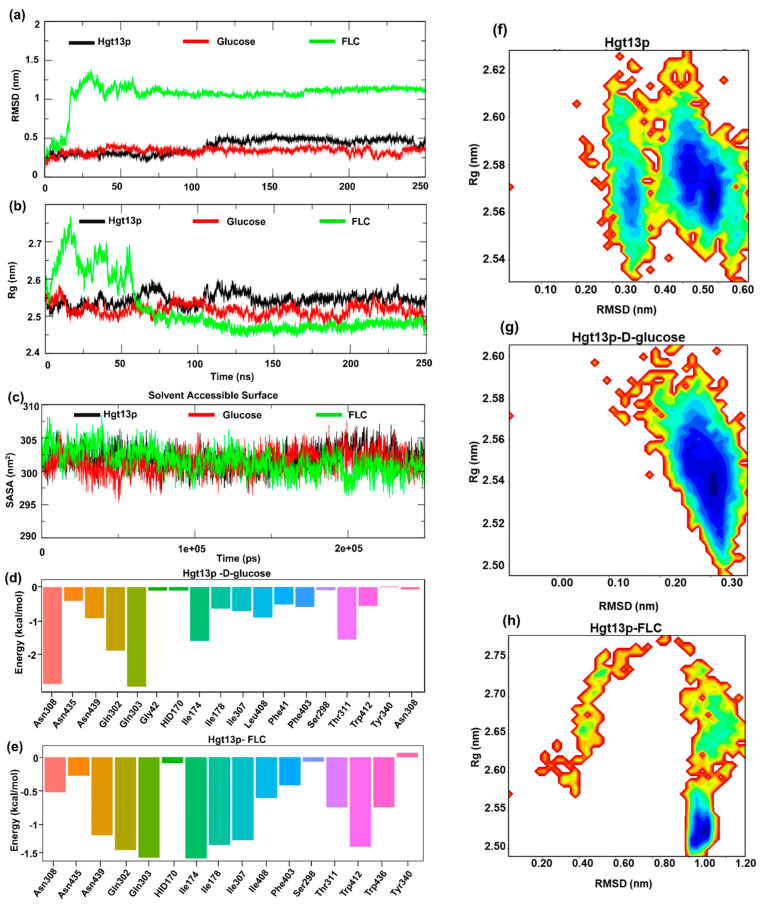
MD simulation of *HGT13* protein with D-glucose and FLC. (**a**) RMSD of Hgt13p, Hgt13p–D-glucose, and Hgt13p–FLC. (**b**) Rg values of Hgt13p, Hgt13p–D-glucose, and Hgt13p–FLC. (**c**) Solvent accessible surface values of Hgt13p, D-glucose, and FLC. (**d**) The residue decomposition plot (MM-GBSA) representing the binding energy contribution of the active site residues of *HGT*, energetically stabilizing glucose at the binding pockets. (**e**) The residue decomposition plot (MM-GBSA) representing the binding energy contribution of the active site residues of *HGT*, energetically stabilizing the FLC at binding pockets. Free energy landscape using the Rg and RMSD value: (**f**) FEL of the Hgt13p, (**g**) FEL of the Hgt13p–D-glucose, (**h**) FEL of the Hgt13p–FLC. In FEL, the key energy basins (minima) are shown in color blue.

The results of Cα-RMSD show that Hgt13p rapidly stabilized within the initial 10 ns of simulation and remained stable thereafter, with an average RMSD 0.47 ± 0.10 nm (Figure 4a). The Hgt13p–D-glucose complex reached equilibrium around the initial ~20 ns and remained stable up to 250 ns, whereas the Hgt13p-FLC complex exhibited initial fluctuations before achieving a stable equilibrium at ~80 ns and was observed to be consistent till the end of the simulation at 250 ns, reflecting conformational adjustment during ligand accommodation. Another parameter, the radius of gyration (Rg), revealed compact structural conformations for both Hgt13p and the Hgt13p–D-glucose complex [53], with average Rg values of 2.54 ± 0.01 nm and 2.51 ± 0.01 nm, respectively (Figure 4b). The Rg trajectory of the Hgt13p-FLC complex stabilized around ~75 ns and remained quite stable thereafter, indicating that the protein undergoes structural rearrangements to spatially accommodate the FLC within the binding site. Similarly, the SASA analysis revealed stable structural dynamics for all three systems (Hgt13p, Hgt13p–D-glucose, and Hgt13p-FLC), with values remaining consistently stabilized around ~302 ± 2 nm^2^ throughout the 250 ns simulation (Figure 4c). This indicates that the overall surface exposure and structural compactness of the protein remained well preserved during the simulation [53,54].

#### 3.7.2. Spatial Stability of Ligands Within the Active Site of Hgt13p

To assess the spatial stability of the ligands within the active site of Hgt13p, we also analysed the RMSD of ligands, D-glucose, and FLC with respect to their initial docked conformations during the simulation period of 250 ns (Figure 4d) [47,54]. Results show that D-glucose remained stable (~0.025 nm) up to 150 ns, followed by a shift to ~0.125 nm, indicating a minor conformational reorientation before stabilizing until 250 ns. In contrast, FLC shows a consistent RMSD (~0.075 nm) throughout the simulation, reflecting better spatial stability of FLC within the Hgt13p active site as compared to D-glucose. Moreover, Figure 4d reveals that the Hgt13p–D-glucose complex is largely stabilized by three to six H-bonds interactions during the simulation, whereas only one H-bond interaction was observed to be consistent with FLC during the simulation, which indicates the limited contribution of polar interactions in spatially stabilizing FLC within the active site of Hgt13p (Figure 4e). These results were observed to be consistent with molecular docking.

#### 3.7.3. Free Energy Landscape of Hgt13p and Docked Complexes

Furthermore, to characterize the conformational dynamics and stability of Hgt13p and docked complexes with D-glucose and FLC, a two-dimensional free energy landscape (FEL) was constructed using the RMSD and Rg as collective variables (Figure 4f–h) [55,56,57]. As shown in Figure 4f, Hgt13p exhibited two major low-energy basins, centred approximately on the Rg value ~2.55–2.57 nm and RMSD ~0.35 nm and ~2.55–2.59 nm and RMSD ~0.50–0.58 nm, respectively, suggesting multiple stable conformations with minor structural flexibility while retaining overall fold stability. The FEL of the Hgt13p–D-glucose complex displayed a single and confined energy basin (Rg ~2.52–2.56 nm; RMSD ~0.28–0.33 nm), indicating a stable conformation of protein–ligand complex (Figure 4g). In contrast, the Hgt13p-FLC complex (Figure 4h) showed a single and dominant energy basin (Rg ~2.50–2.55 nm; RMSD ~1.00–1.10 nm), consistent with RMSD results and confirming the stable binding of FLC to Hgt13p.

#### 3.7.4. Binding Free Energy of Ligands

Finally, to quantitatively evaluate the binding affinities and energetic stability of ligand association with Hgt13p, MM-GBSA calculations were performed using equilibrated snapshots from the 250 ns MD trajectories [58]. The total binding free energy (ΔG_bind_) was decomposed into van der Waals (ΔE_vdW_), electrostatic (ΔE_EEL_), polar solvation (ΔG_GB_), and nonpolar solvation (ΔG_SURF_) components to elucidate the key energetic contributors to complex stability. The Hgt13p–D-glucose complex exhibited a favourable binding free energy of ΔG_bind_ = −23.57 ± 4.67 kcal/mol, primarily driven by strong electrostatic interactions (ΔE_EEL_ = −45.07 ± 7.20 kcal/mol) and supported by van der Waals forces (ΔE_vdW_ = −20.37 ± 2.70 kcal/mol), which together compensated for the desolvation penalty from polar solvation. Consistent with the molecular docking results, D-glucose shows stable polar interactions with residues Gln303 and Asn308, while Gln302 and Ile174 contributed moderately to stabilizing the ligand at the active site (Figure 4d).

Differently, the Hgt13p-FLC complex showed the major contribution of energetic term ΔE_vdW_ − 41.72 ± 2.39 kcal/mol followed by ΔE_EEL_ − 19.81 ± 4.17 kcal/mol, resulting in an overall binding free energy ΔG_bind_ − 34.03 ± 3.63 kcal/mol. FLC shows stable hydrophobic interaction with Ile174, Ile178, and Trp412with Gln302 and Gln303, complemented by polar interaction with Gln302 and Gln303. Additionally, Asn439 and Ile307 contributed moderate polar and hydrophobic interactions, respectively, in stabilizing the complex, which was observed consistent with molecular docking (Figure 4e).

Taken together, the MD simulation and MM-GBSA analyses corroborated the molecular docking results, revealing that both ligands bind favourably to Hgt13p. However, FLC exhibited a stronger binding affinity and higher complex stability than D-glucose, consistent with its stable MD trajectory and compact FEL profile.

## 4. Discussion

Among the various strategies employed by *Candida* species, including *C. auris*, enhanced drug efflux mediated by primary ABC and MFS transporters remains the most well-established mechanism of antifungal resistance [18,59]. Although amphiphilic drugs are generally believed to enter cells through passive diffusion, recent evidence suggests that carrier-mediated drug import, resulting in altered intracellular accumulation, also contributes to resistance [27]. Notably, no dedicated drug importer has yet been identified. In this context, HGTs have emerged as potential candidates capable of facilitating drug uptake through a ‘piggyback’ mechanism. Our findings reinforce this emerging concept by demonstrating that HGTs can modulate drug uptake and consequently influence the development of antifungal resistance in *C. auris*.

We identified 15 putative HGT genes in *C. auris*, and phylogenetic clustering suggests that *C. auris* HGTs are more closely related to those of *C. albicans*, indicating conserved structural and functional roles within the CTG clade [60,61] and reflecting essential roles of sugar transport systems in metabolic adaptation and environmental sensing in *C.* species [62,63,64]. Our findings also indicate that the HGT domain is conserved across all 15 identified transporters despite notable variation in protein size. The basal transcriptional activity of most HGT genes under basal conditions indicates that some transporters are actively involved in carbohydrate acquisition. Their altered transcriptional expression upon FLC exposure further indicates broader physiological roles that extend beyond sugar uptake. This aligns with recent reports suggesting that sugar transport systems can influence cellular homeostasis, stress responses, and membrane energetic factors that collectively impact antifungal susceptibility [65,66].

**Figure 5:**
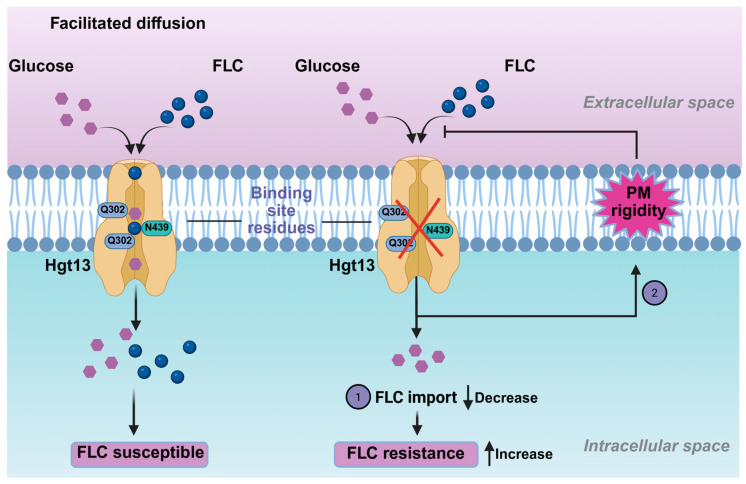
Schematic overview of the pathway contributing to FLC resistance in *C. auris*. High binding affinity of FLC to Hgt13p supports a “piggyback” drug uptake mechanism. In contrast, loss of the *HGT13* membrane transporter alters membrane rigidity, and together these pathways enhance FLC resistance in *C. auris*.

Functional analysis of the mini-library of HGT deletions highlighted the specific importance of the *HGT13* gene in modulating antifungal susceptibility. Notably, Δ*hgt13* showed a 4-fold increase in FLC resistance, a phenotype not observed in the other tested HGT mutants. This increased resistance was accompanied by decreased intracellular ^3^H-FLC accumulation, demonstrating that the Δ*hgt13* mutant cells internalised less FLC than the wild-type ones. Recent studies in *Candida* species have identified HGT transporters as contributors to drug import, with their deletion leading to increased drug resistance [28,33]. In contrast, glucose accumulation assays in the Δ*hgt13* mutant showed no significant change, likely due to compensation by the remaining 14 HGT genes in *C. auris*. This aligns with findings in *C. albicans*, where the *HGT13* ortholog was also found to be dispensable for glucose transport [28,67]. The Δ*hgt13* mutant was also found to impact plasma membrane homeostasis, as was evident from NPN uptake assays, which showed increased membrane rigidity. The combined phenotype, characterised by enhanced resistance along with reduced intracellular drug levels and increased membrane rigidity, supports the hypothesis that *HGT13* could impact FLC-import-associated processes (Figure 5).

Furthermore, the computational analysis substantiates the experimental evidence of a functional link between Hgt13p and FLC import. Molecular docking revealed that FLC binds to the key residues of Hgt13p, which overlap with the molecular interactions of D-glucose with the XylE protein [40]. Importantly, MD simulations provided a dynamic characterization of these interactions, revealing their stability and conformational behaviours beyond the static docking predictions [53]. Throughout the simulation, FLC remained consistently engaged within the Hgt13p binding pocket, maintaining stable interactions with key conserved residues, whereas D-glucose exhibited more transient positioning. Moreover, the higher binding free energy calculated for FLC relative to D-glucose further indicates its more stable association with Hgt13p. Together, these computational analyses provide a structural interpretation of the molecular interactions of FLC and D-glucose with Hgt13p, aligning with the experimental observation that *HGT13* contributes to FLC import and influences susceptibility. The preferential and tighter binding of FLC to Hgt13p compared to D-glucose raises the possibility that azoles may exploit conserved HGT scaffolds as opportunistic entry points, a concept with implications for understanding intrinsic and acquired resistance. Thus, these findings expand the functional repertoire of HGT, highlighting its role as a modulator of antifungal susceptibility beyond its conventional role in nutrient transport.

## 5. Conclusions

In conclusion, this study systematically identifies and characterizes 15 putative sugar transporter genes in *C. auris*, establishing their evolutionary conservation with *C. albicans* orthologs through phylogenetic and homology analyses. Integrative structural modeling revealed conserved MFS transporter architecture alongside divergence in transmembrane helices, indicative of functional specialization. Expression profiling demonstrated distinct basal and FLC responsive transcriptional patterns, with several HGTs being transcriptionally repressed upon drug exposure. Functional analysis highlighted *HGT13* as a critical determinant of FLC susceptibility, where its deletion conferred a four-fold increase in FLC resistance, reduced intracellular ^3^H-FLC accumulation, and enhanced membrane rigidity without compromising glucose uptake. In silico docking and molecular dynamics simulations further demonstrated that Hgt13p accommodates both glucose and FLC within overlapping binding pockets, mediated by conserved residues (Gln302, Gln303, and Asn308) with a higher binding affinity for FLC. Collectively, these findings uncover *HGT13* as a previously unrecognized FLC importer that links sugar transport, membrane dynamics, and antifungal susceptibility in *C. auris*. Targeting this transporter thus represents a promising therapeutic avenue to counteract multidrug resistance in this priority fungal pathogen.

## Figures and Tables

**Table 1:** List of the constructed knockout mutants of HGT genes in *C. auris* wild-type strain CBS10913T.

Strains/Mutants	Gene Name and ID
CBS10913T (wild type)	
CBS10913T_Δ*hgt2*	*HGT2* (B9J08_005568)
CBS10913T_Δ*hgt12*	*HGT12* (B9J08_002250)
CBS10913T_Δ*hgt13*	*HGT13* (B9J08_001015)
CBS10913T_Δ*hgt19*	*HGT19* (B9J08_005025)
CBS10913T_Δ*hxt5*	*HXT5* (B9J08_002646)
CBS10913T_Δ*hgt4*	*HGT4* (B9J08_002259)

## Data Availability

All the data are available in the manuscript and supplementary files.

## Supplementary Materials

The following supporting information can be downloaded at: https://www.mdpi.com/article/10.3390/jof12020094/s1, Figure S1: Conserved sugar transporter domain and predicted 3D structure of sugar transporters; Figure S2: Structural alignment of Hgt13p and XylE; Figure S3: Multiple sequence alignment of 15 putative sugar transporter proteins along with the *E. coli* XylE protein; Table S1: *C. auris* sugar transporter genes: BLAST percentage identity against *C. albicans* and *S. cerevisiae*; Table S2: List of oligonucleotides used for the RT-PCR; Table S3: List of oligonucleotides used for the construction of the gene knockout.

## References

[1] Kim J.-Y. (2016). Human Fungal Pathogens: Why Should We Learn?. J. Microbiol..

[2] Schelenz S., Hagen F., Rhodes J.L., Abdolrasouli A., Chowdhary A., Hall A., Ryan L., Shackleton J., Trimlett R., Meis J.F. (2016). First Hospital Outbreak of the Globally Emerging *Candida auris* in a European Hospital. Antimicrob. Resist. Infect. Control.

[3] Forsberg K., Woodworth K., Walters M., Berkow E.L., Jackson B., Chiller T., Vallabhaneni S. (2019). *Candida auris*: The Recent Emergence of a Multidrug-Resistant Fungal Pathogen. Med. Mycol..

[4] Anuradha Chowdhary A.C., Anupam Prakash A.P., Cheshta Sharma C.S., Kordalewska M., Anil Kumar A.K., Smita Sarma S.S., Bansidhar Tarai B.T., Ashutosh Singh A.S., Gargi Upadhyaya G.U., Shalini Upadhyay S.U. (2018). A Multicentre Study of Antifungal Susceptibility Patterns among 350 *Candida auris* Isolates (2009–17) in India: Role of the ERG11 and FKS1 Genes in Azole and Echinocandin Resistance. J. Antimicrob. Chemother..

[5] Jacobs S.E., Jacobs J.L., Dennis E.K., Taimur S., Rana M., Patel D., Gitman M., Patel G., Schaefer S., Iyer K. (2022). *Candida auris* Pan-Drug-Resistant to Four Classes of Antifungal Agents. Antimicrob. Agents Chemother..

[6] Carolus H., Pierson S., Muñoz J.F., Subotic A., Cruz R.B., Cuomo C.A., Van Dijck P. (2021). Genome-wide analysis of experimentally evolved *Candida auris* reveals multiple novel mechanisms of multidrug resistance. mBio.

[7] Wasi M., Kumar Khandelwal N., Moorhouse A.J., Nair R., Vishwakarma P., Bravo Ruiz G., Ross Z.K., Lorenz A., Rudramurthy S.M., Chakrabarti A. (2019). ABC Transporter Genes Show Upregulated Expression in Drug-Resistant Clinical Isolates of *Candida auris*: A Genome-Wide Characterization of ATP-Binding Cassette (ABC) Transporter Genes. Front. Microbiol..

[8] Zhou W., Li X., Lin Y., Yan W., Jiang S., Huang X., Yang X., Qiao D., Li N. (2021). A Comparative Transcriptome between Anti-Drug Sensitive and Resistant *Candida auris* in China. Front. Microbiol..

[9] Narayanan A. (2022). Directed Evolution Detects Supernumerary Centric Chromosomes Conferring Resistance to Azoles in *Candida auris*. mBio.

[10] Shivarathri R., Jenull S., Chauhan M., Singh A., Mazumdar R., Chowdhary A., Kuchler K., Chauhan N. (2022). Comparative Transcriptomics Reveal Possible Mechanisms of Amphotericin B Resistance in *Candida auris*. Antimicrob. Agents Chemother..

[11] Massic L., Doorley L.A., Jones S.J., Richardson I., Siao D.D., Siao L., Dykema P., Hua C., Schneider E., Cuomo C.A. (2025). Acquired Amphotericin B Resistance Attributed to a Mutated ERG3 in Candidozyma Auris. Antimicrob. Agents Chemother..

[12] Rybak J.M., Barker K.S., Muñoz J.F., Parker J.E., Ahmad S., Mokaddas E., Abdullah A., Elhagracy R.S., Kelly S.L., Cuomo C.A. (2022). In Vivo Emergence of High-Level Resistance during Treatment Reveals the First Identified Mechanism of Amphotericin B Resistance in *Candida auris*. Clin. Microbiol. Infect..

[13] Chauhan A., Carolus H., Sofras D., Kumar M., Kumar P., Nair R., Narayanan A., Yadav K., Ali B., Biriukov V. (2025). Multi-Omics Analysis of Experimentally Evolved *Candida auris* Isolates Reveals Modulation of Sterols, Sphingolipids, and Oxidative Stress in Acquired Amphotericin B Resistance. Mol. Microbiol..

[14] Phan-Canh T., Nguyen-Le D.-M., Luu P.-L., Khunweeraphong N., Kuchler K. (2025). Rapid in Vitro Evolution of Flucytosine Resistance in *Candida auris*. mSphere.

[15] LaFleur M.D., Kumamoto C.A., Lewis K. (2006). *Candida albicans* Biofilms Produce Antifungal-Tolerant Persister Cells. Antimicrob. Agents Chemother..

[16] Iyer K.R., Robbins N., Cowen L.E. (2022). The Role of *Candida albicans* Stress Response Pathways in Antifungal Tolerance and Resistance. iScience.

[17] Patra S., Raney M., Pareek A., Kaur R. (2022). Epigenetic Regulation of Antifungal Drug Resistance. J. Fungi.

[18] Cowen L.E., Sanglard D., Howard S.J., Rogers P.D., Perlin D.S. (2015). Mechanisms of Antifungal Drug Resistance. Cold Spring Harb. Perspect. Med..

[19] Perlin D.S., Rautemaa-Richardson R., Alastruey-Izquierdo A. (2017). The Global Problem of Antifungal Resistance: Prevalence, Mechanisms, and Management. Lancet Infect. Dis..

[20] Rybak J.M., Doorley L.A., Nishimoto A.T., Barker K.S., Palmer G.E., Rogers P.D. (2019). Abrogation of Triazole Resistance upon Deletion of CDR1 in a Clinical Isolate of *Candida auris*. Antimicrob. Agents Chemother..

[21] Prasad R. (1995). Molecular Cloning and Characterization of a Novel Gene of *Candida albicans*, CDR1, Conferring Multiple Resistance to Drugs and Antifungals. Curr. Genet..

[22] Sun N., Li D., Fonzi W., Li X., Zhang L., Calderone R. (2013). Multidrug-Resistant Transporter Mdr1p-Mediated Uptake of a Novel Antifungal Compound. Antimicrob. Agents Chemother..

[23] Rybak J.M., Muñoz J.F., Barker K.S., Parker J.E., Esquivel B.D., Berkow E.L., Lockhart S.R., Gade L., Palmer G.E., White T.C. (2020). Mutations in *TAC1B*: A Novel Genetic Determinant of Clinical Fluconazole Resistance in *Candida auris*. mBio.

[24] Rajesh-Khanna D.-S., Piña Páez C.G., He S., Dolan E.G., Mirpuri K.S., Stajich J.E., Hogan D.A. (2025). Coordinated Regulation of Mdr1- and Cdr1-Mediated Protection from Antifungals by the Mrr1 Transcription Factor in Emerging *Candida* spp.. mBio.

[25] Mansfield B.E. (2010). Azole Drugs Are Imported by Facilitated Diffusion in *Candida albicans* and Other Pathogenic Fungi. PLoS Pathog..

[26] Esquivel B.D., Smith A.R., Zavrel M., White T.C. (2015). Azole Drug Import into the Pathogenic Fungus *Aspergillus fumigatus*. Antimicrob. Agents Chemother..

[27] Galocha M., Costa I.V., Teixeira M.C. (2020). Carrier-mediated drug uptake in fungal pathogens. Genes.

[28] Galocha M. (2022). Genomic Evolution towards Azole Resistance in *Candida glabrata* Clinical Isolates Unveils the Importance of CgHxt4/6/7 in Azole Accumulation. Commun. Biol..

[29] Yasin R., Usman S., Qin Q., Gong X., Wang B., Wang L., Jin C., Fang W. (2025). Key Sugar Transporters Drive Development and Pathogenicity in *Aspergillus flavus*. Front. Cell. Infect. Microbiol..

[30] Donzella L., Sousa M.J., Morrissey J.P. (2023). Evolution and Functional Diversification of Yeast Sugar Transporters. Essays Biochem..

[31] Redhu A.K., Shah A.H., Prasad R. (2016). MFS Transporters of *Candida* Species and Their Role in Clinical Drug Resistance. FEMS Yeast Res..

[32] Rubio-Texeira M., Van Zeebroeck G., Voordeckers K., Thevelein J.M. (2010). *Saccharomyces cerevisiae* Plasma Membrane Nutrient Sensors and Their Role in PKA Signaling. FEMS Yeast Res..

[33] Biswas C. (2013). Functional Characterization of the Hexose Transporter Hxt13p: An Efflux Pump That Mediates Resistance to Miltefosine in Yeast. Fungal Genet. Biol..

[34] Nourani A., Wesolowski-Louvel M., Delaveau T., Jacq C., Delahodde A. (1997). Multiple-Drug-Resistance Phenomenon in the Yeast *Saccharomyces cerevisiae*: Involvement of Two Hexose Transporters. Mol. Cell. Biol..

[35] Zamith-Miranda D., Heyman H.M., Cleare L.G., Couvillion S.P., Clair G.C., Bredeweg E.L., Gacser A., Nimrichter L., Nakayasu E.S., Nosanchuk J.D. (2019). Multi-Omics Signature of *Candida auris*, an Emerging and Multidrug-Resistant Pathogen. mSystems.

[36] Gietz D., Jean A.S., Woods R.A., Schiestl R.H. (1992). Improved Method for High Efficiency Transformation of Intact Yeast Cells. Nucleic Acids Res..

[37] (2002). Reference Method for Broth Dilution Antifungal Susceptibility Testing of Yeasts; Approved Standard 2002.

[38] Esquivel B.D., White T.C. (2017). Accumulation of Azole Drugs in the Fungal Plant Pathogen *Magnaporthe oryzae* Is the Result of Facilitated Diffusion Influx. Front. Microbiol..

[39] Niemirowicz K., Durnaś B., Tokajuk G., Piktel E., Michalak G., Gu X., Kułakowska A., Savage P.B., Bucki R. (2017). Formulation and Candidacidal Activity of Magnetic Nanoparticles Coated with Cathelicidin LL-37 and Ceragenin CSA-13. Sci. Rep..

[40] Sun L., Zeng X., Yan C., Sun X., Gong X., Rao Y., Yan N. (2012). Crystal Structure of a Bacterial Homologue of Glucose Transporters GLUT1–4. Nature.

[41] Bugnon M., Röhrig U.F., Goullieux M., Perez M.A., Daina A., Michielin O., Zoete V. (2024). SwissDock 2024: Major Enhancements for Small-Molecule Docking with Attracting Cavities and AutoDock Vina. Nucleic Acids Res..

[42] Eberhardt J., Santos-Martins D., Tillack A.F., Forli S. (2021). AutoDock Vina 1.2.0: New Docking Methods, Expanded Force Field, and Python Bindings. J. Chem. Inf. Model..

[43] Van Der Spoel D., Lindahl E., Hess B., Groenhof G., Mark A.E., Berendsen H.J.C. (2005). GROMACS: Fast, Flexible, and Free. J. Comput. Chem..

[44] Khan H.M., MacKerell A.D., Reuter N. (2019). Cation-π Interactions between Methylated Ammonium Groups and Tryptophan in the CHARMM36 Additive Force Field. J. Chem. Theory Comput..

[45] Vanommeslaeghe K., MacKerell A.D. (2012). Automation of the CHARMM General Force Field (CGenFF) I: Bond Perception and Atom Typing. J. Chem. Inf. Model..

[46] Feng S., Park S., Choi Y.K., Im W. (2023). CHARMM-GUI *Membrane Builder*: Past, Current, and Future Developments and Applications. J. Chem. Theory Comput..

[47] Mishra C.B., Pandey P., Sharma R.D., Malik M.Z., Mongre R.K., Lynn A.M., Prasad R., Jeon R., Prakash A. (2021). Identifying the Natural Polyphenol Catechin as a Multi-Targeted Agent against SARS-CoV-2 for the Plausible Therapy of COVID-19: An Integrated Computational Approach. Brief. Bioinform..

[48] Valdés-Tresanco M.S., Valdés-Tresanco M.E., Valiente P.A., Moreno E. (2021). gmx_MMPBSA: A New Tool to Perform End-State Free Energy Calculations with GROMACS. J. Chem. Theory Comput..

[49] Cauldron N.C., Shea T., Cuomo C.A. (2024). Improved Genome Assembly of *Candida auris* Strain B8441 and Annotation of B11205. Microbiol. Resour. Announc..

[50] Structural Advances for the Major Facilitator Superfamily (MFS) Transporters—PubMed. https://pubmed.ncbi.nlm.nih.gov/23403214/.

[51] Quistgaard E.M., Löw C., Guettou F., Nordlund P. (2016). Understanding Transport by the Major Facilitator Superfamily (MFS): Structures Pave the Way. Nat. Rev. Mol. Cell Biol..

[52] Abramson J., Adler J., Dunger J., Evans R., Green T., Pritzel A., Ronneberger O., Willmore L., Ballard A.J., Bambrick J. (2024). Accurate Structure Prediction of Biomolecular Interactions with AlphaFold 3. Nature.

[53] Karplus M., McCammon J.A. (2002). Molecular Dynamics Simulations of Biomolecules. Nat. Struct. Biol..

[54] Gorelov S., Titov A., Tolicheva O., Konevega A., Shvetsov A. (2024). DSSP in GROMACS: Tool for Defining Secondary Structures of Proteins in Trajectories. J. Chem. Inf. Model..

[55] Moritsugu K., Terada T., Kidera A. (2017). Free-Energy Landscape of Protein–Ligand Interactions Coupled with Protein Structural Changes. J. Phys. Chem. B.

[56] Stenström O., Diehl C., Modig K., Akke M. (2024). Ligand-Induced Protein Transition State Stabilization Switches the Binding Pathway from Conformational Selection to Induced Fit. Proc. Natl. Acad. Sci. USA.

[57] Ahmad S., Naiyer A., Kumar P., Parkash A. (2024). Mechanism of Folding and Stability of Met80Gly Mutant of Cytochrome-c. J. Mol. Liq..

[58] Genheden S., Ryde U. (2015). The MM/PBSA and MM/GBSA Methods to Estimate Ligand-Binding Affinities. Expert Opin. Drug Discov..

[59] Sanglard D., Coste A.T. (2016). Activity of Isavuconazole and Other Azoles against Candida Clinical Isolates and Yeast Model Systems with Known Azole Resistance Mechanisms. Antimicrob. Agents Chemother..

[60] Butler G., Rasmussen M.D., Lin M.F., Santos M.A., Sakthikumar S., Munro C.A., Rheinbay E., Grabherr M., Forche A., Reedy J.L. (2009). Evolution of Pathogenicity and Sexual Reproduction in Eight Candida Genomes. Nature.

[61] Jackson A.P., Gamble J.A., Yeomans T., Moran G.P., Saunders D., Harris D., Aslett M., Barrell J.F., Butler G., Citiulo F. (2009). Comparative Genomics of the Fungal Pathogens Candida Dubliniensis and Candida Albicans. Genome Res..

[62] Qadri H., Qureshi M.F., Mir M.A., Shah A.H. (2021). Glucose—The X Factor for the Survival of Human Fungal Pathogens and Disease Progression in the Host. Microbiol. Res..

[63] Van Ende M., Wijnants S., Van Dijck P. (2019). Sugar Sensing and Signaling in *Candida albicans* and *Candida glabrata*. Front. Microbiol..

[64] Chattopadhyay A., Singh R., Das A.K., Maiti M.K. (2020). Characterization of Two Sugar Transporters Responsible for Efficient Xylose Uptake in an Oleaginous Yeast *Candida tropicalis* SY005. Arch. Biochem. Biophys..

[65] Rutherford J.C., Bahn Y.-S., van den Berg B., Heitman J., Xue C. (2019). Nutrient and Stress Sensing in Pathogenic Yeasts. Front. Microbiol..

[66] Rodaki A., Bohovych I.M., Enjalbert B., Young T., Odds F.C., Gow N.A.R., Brown A.J.P. (2009). Glucose Promotes Stress Resistance in the Fungal Pathogen *Candida albicans*. Mol. Biol. Cell.

[67] Leandro M.J., Gonçalves P., Spencer-Martins I. (2006). Two Glucose/Xylose Transporter Genes from the Yeast Candida Intermedia: First Molecular Characterization of a Yeast Xylose–H+ Symporter. Biochem. J..

