# Supplementary material for: Comprehensive Insights into Sugar Transporters of Candidozyma auris and Their Roles in Antifungal Resistance
Source: J Fungi (Basel). 2026 Jan 30;12(2):94. doi: 10.3390/jof12020094 (PMC12942029; doi:10.3390/jof12020094)
Supplement: Supplementary file 1 [file jof-12-00094-s001.zip › jof-4096789-supplementary.pdf]

Supplementary figures and tables

(a)

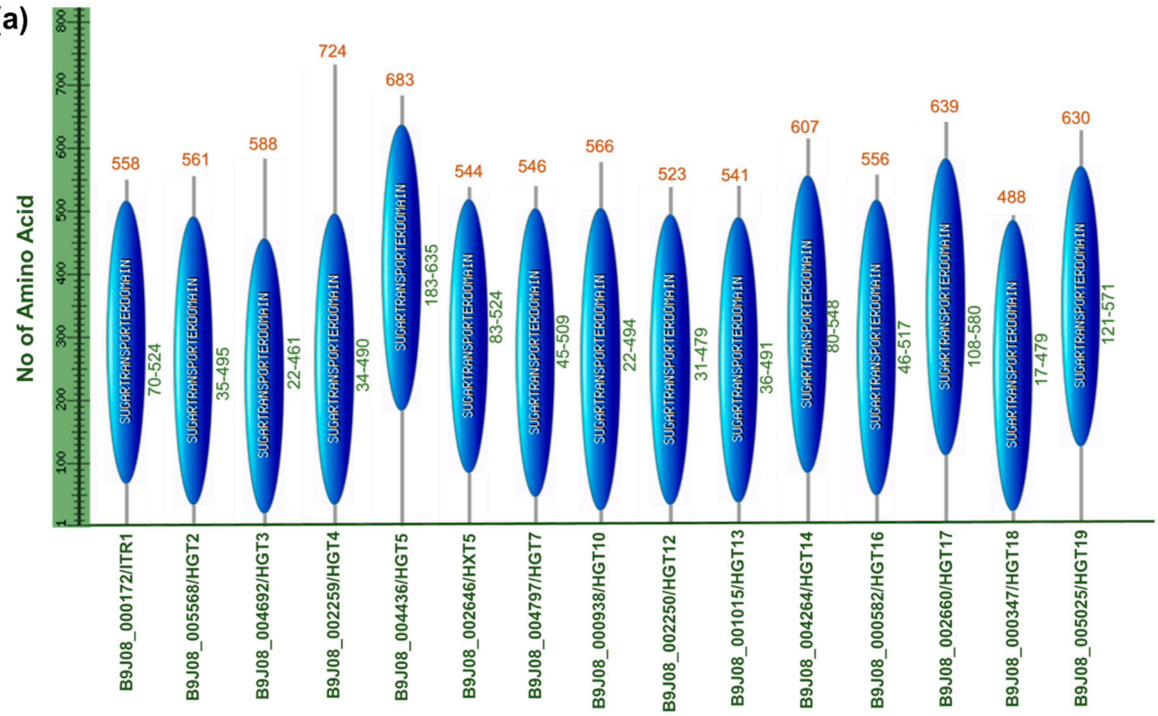

(b)

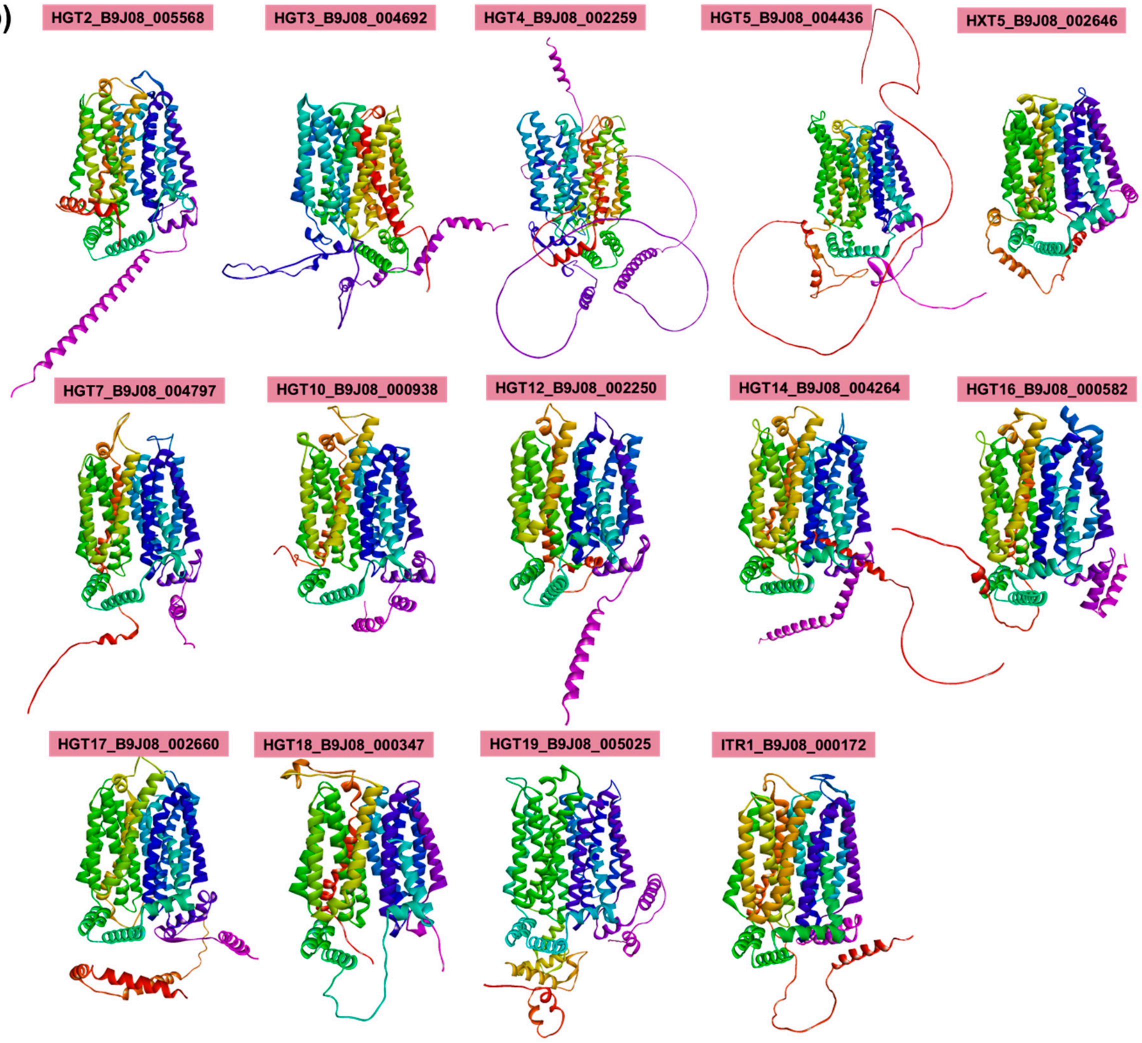

Supplementary Figure S1: a. Conserved sugar transporter domain of different size of 14 sugar transporter genes. **b.** 3D structure of the 14 putative sugar transporter generated by AlphaFold3 server.

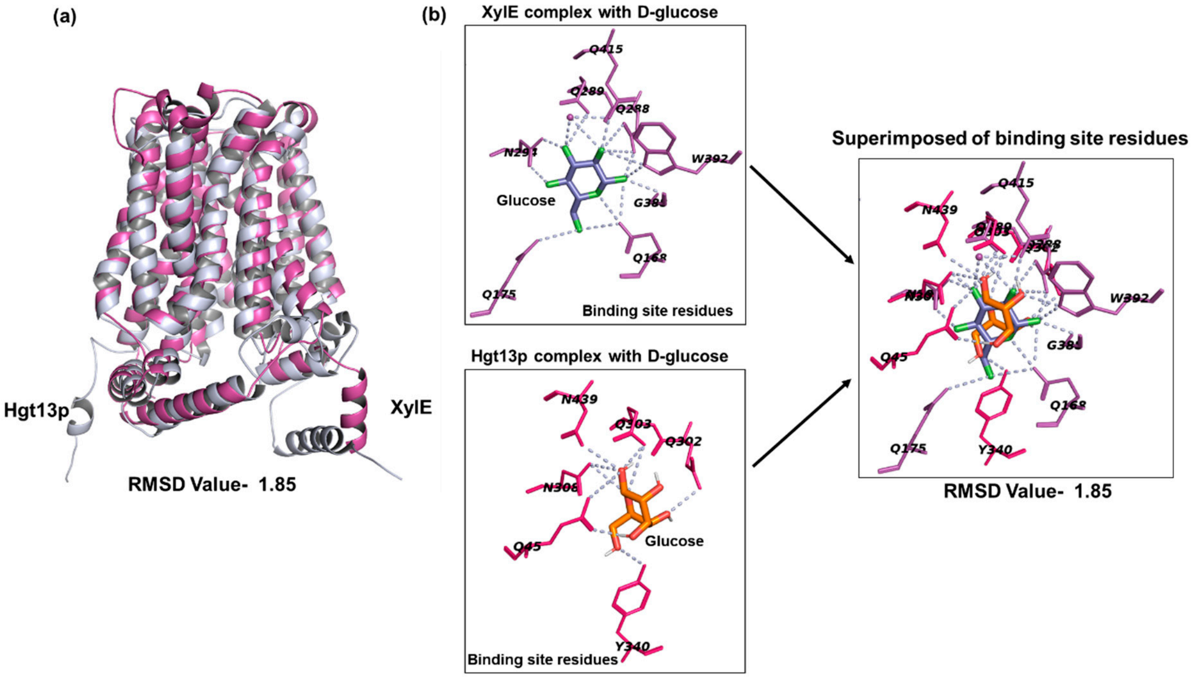

Supplementary Figure S2: Structural alignment of Hgt13p and XylE. a. The structural alignment was generated of Hgt13p using XylE as the reference protein, revealing an RMSD of 1.85, indicating a high degree of structural similarity. **b.** Binding-site residues of both proteins was superimposed, highlighting the similarity of their binding pockets.

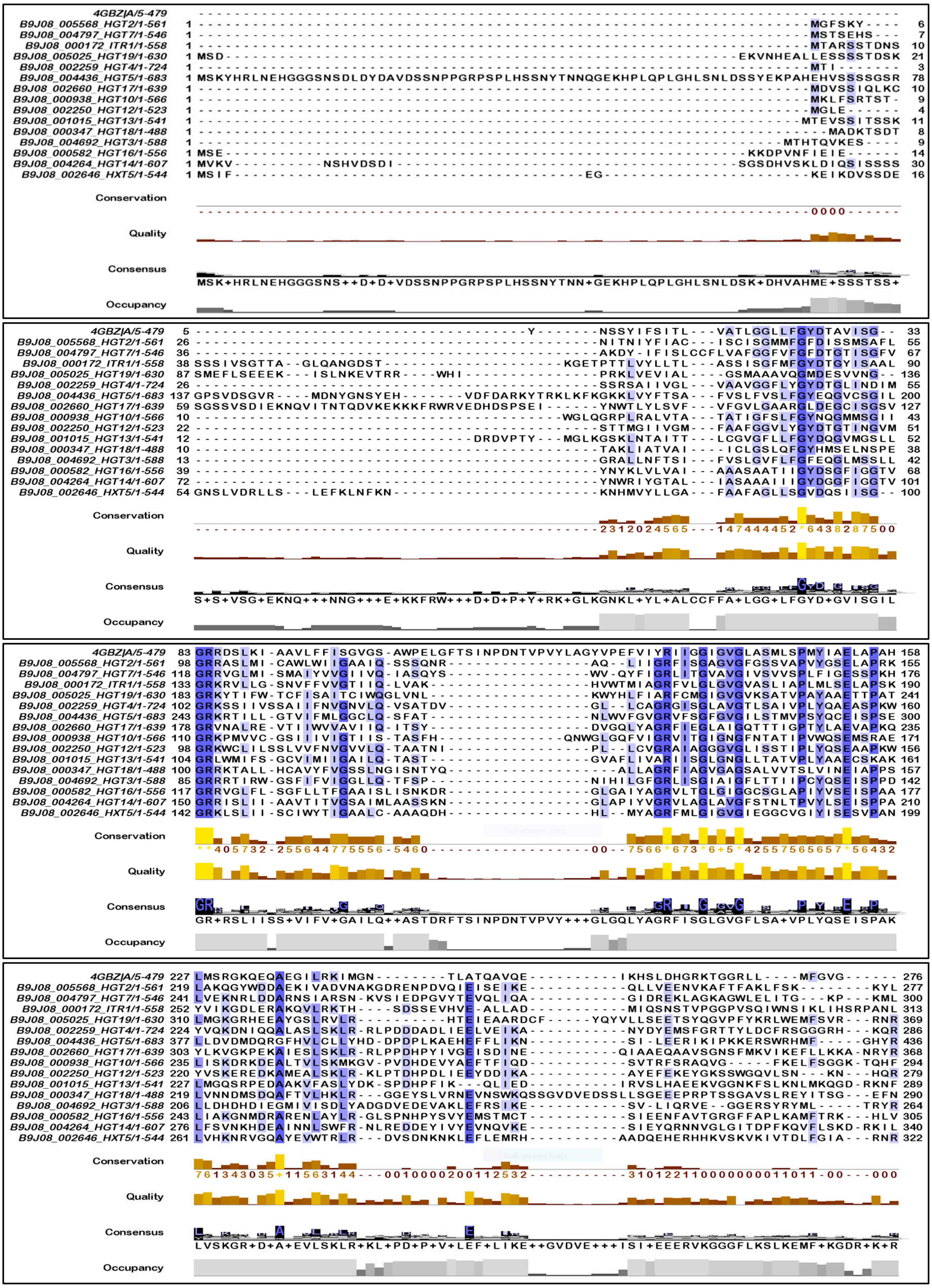
4GBZ|A/5-479
B9J08_005568_HGT2/1-561
B9J08_004797_HGT7/1-546
B9J08_000172_ITR1/1-558
B9J08_005025_HGT19/1-630
B9J08_002259_HGT4/1-724
B9J08_004436_HGT5/1-683
B9J08_002660_HGT17/1-639
B9J08_000938_HGT10/1-566
B9J08_002250_HGT12/1-523
B9J08_001015_HGT13/1-541
B9J08_000347_HGT18/1-488
B9J08_004692_HGT3/1-588
B9J08_000582_HGT16/1-556
B9J08_004264_HGT14/1-607
B9J08_002646_HXT5/1-544
Conservation
Quality
Consensus
Occupancy

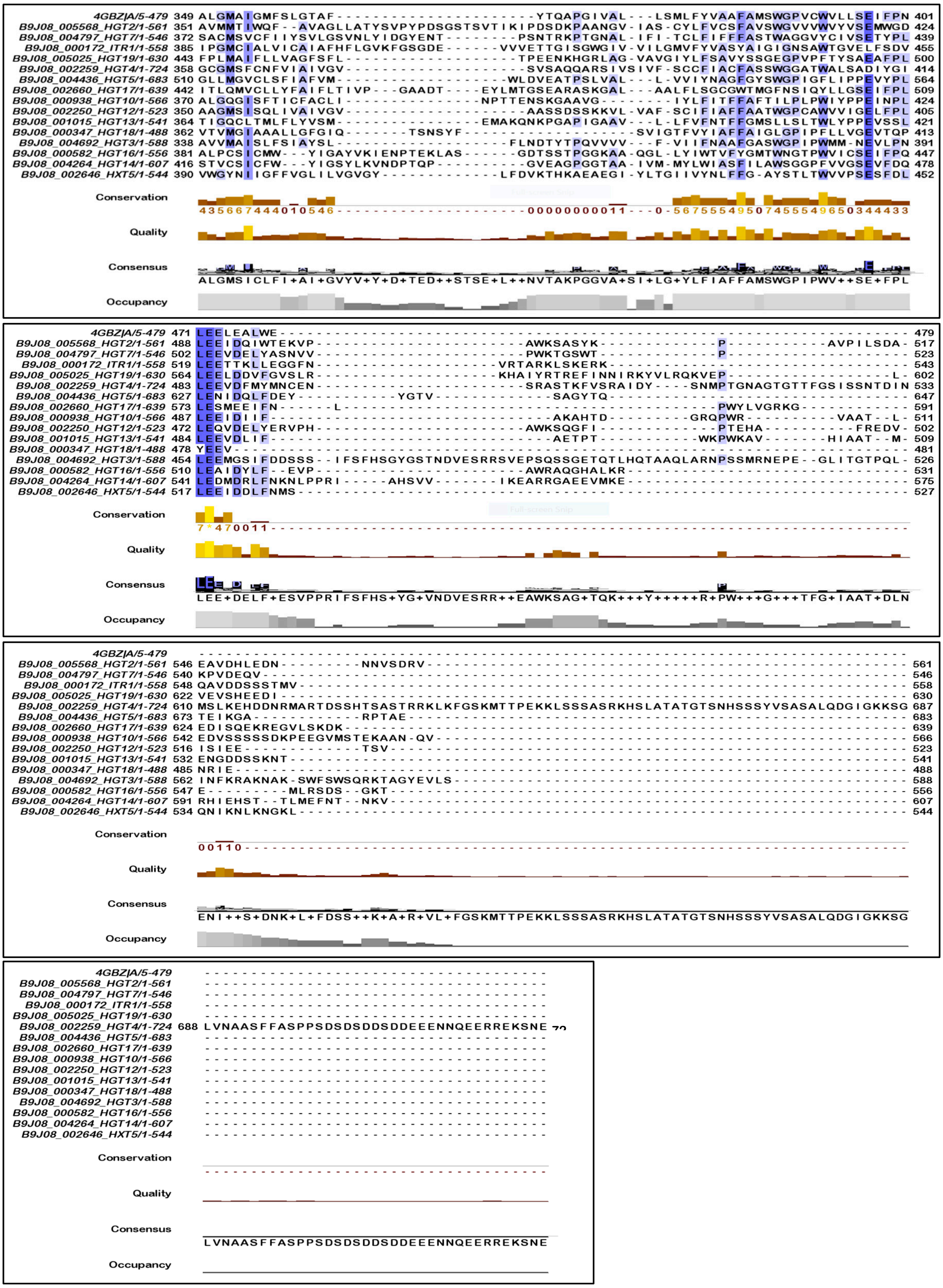

Supplementary Figure S3: Multiple sequence alignment of 15 putative sugar transporter proteins along with the *E. coli* XylE protein

Supplementary Table S1: List of *C. auris* sugar transporter genes. Find the percentage identity with *C. albicans* and *S. cerevisiae* by using the blast

***C. auris* gene name/id**	***C. albicans* orthologs gene name**	**Identity %**	***S. Cerevisiae* orthologs gene name**	**Identity %**	**Gene Function**
HGT2 (B9J08_005568)	HGT1<br>HGT2	66.10%<br>66.20%	HXT2	33.10%	Putative MFS glucose transporter; expressed in rich medium with 2% glucose
HGT7 (B9J08_004797)	HGT6<br>HGT7<br>HGT8	66.70%<br>65.70%<br>67.30%	HXT7<br>HXT6<br>HXT4	61.20%<br>61.20%<br>59.20%	Putative MFS glucose transporter; glucose, fluconazole, Snf3 induced, expressed at high glucose
ITR1 (B9J08_000172)	ITR1	70.25%	ITR1<br>ITR2	50.30%<br>52.10%	MFS inositol transporter; uptake of exogenous inositol; fluconazole, caspofungin repressed
HGT19 (B9J08_005025)	HGT19	70.30%	HXT8	30.20%	Putative MFS glucose/myo-inositol transporter
HGT4 (B9J08_002259)	HGT4	53.20%	SNF3<br>RGT2	56.70%<br>56.20%	Ortholog(s) have role in fructose transmembrane transport, glucose mediated signaling pathway
HGT5(B9J08_004436)	HGT5	55.10%	STL1	31.40%	Putative glucose transporter;

					induced at low glucose
HGT17 (B9J08_002660)	HGT17	60.40%	HXT8	30.80%	Putative MFS family glucose transporter; induced at low (0.2%, compared to 2%) glucose in rich media
HGT10 (B9J08_000938)	HGT10	67.40%	STL1	57.30%	Glycerol permease involved in glycerol uptake
HGT12 (B9J08_002250)	HGT12	62.80%	RGT2<br>SNF3<br>HXT5	47.60%<br>47.80%<br>37.50%	Glucose, fructose, mannose transporter; Snf3p-induced
HGT13 (B9J08_001015)	HGT13	50.10%	STL1	40.10%	Predicted sugar transporter, involved in glycerol utilization; oxidative stress-induced via Cap1p
HGT18 (B9J08_000347)	HGT18	61%	YBR241C	45.50%	Putative glucose transporter; expressed in rich medium with 2% glucose
HGT3 (B9J08_004692)	HGT3	57.20%	STL1	31%	Putative glucose transporter; expressed in rich medium with 2% glucose
HGT16 (B9J08_000582)	HGT16	63.40%	HXT8<br>HXT1	32.80%<br>30.50%	Putative glucose transporter; expressed in rich medium with 2% glucose
HGT14 (B9J08_004264)	HGT14	69.90%	HXT1	28%	Putative MFS glucose transporter; expressed in rich medium with 2% glucose

HXT5 (B9J08_002646)	HXT5	61.90%	HXT3	30.10%	Putative sugar transporter; induced by ciclopirox olamine; Snf3-induced; possibly essential gene

Supplementary Table S2: List of oligonucleotides used for the RT-PCR

Genes ID/Gene Name	Primers	5' - 3' Sequences
**B9J08_000172/ITR1**	Forward Primer	CCCAAGATACTACGTGATTAAGG
	Reverse Primer	AACCCACATGCCAATATAAGAG
**B9J08_005568/HGT2**	Forward Primer	CTCGTGTATATCAGGGATGATG
	Reverse Primer	GAACCAAAGAAAGATCCAAGTG
**B9J08_004692/HGT3**	Forward Primer	ACTATCATTCCCTGCTACCAATC
	Reverse Primer	TTCCGAGCCAAAGCAATAATC
**B9J08_002259/HGT4**	Forward Primer	CCGCAGATATTTACGGTATTAG
	Reverse Primer	TATACGTAGACTACACCCAAAG
**B9J08_004436/HGT5**	Forward Primer	GGCGTCGGTATATTATCCACTATG
	Reverse Primer	TCTCCAATGTCCTGAATGAAGTAG
**B9J08_002646/HXT5**	Forward Primer	CATGGTGTACCTTCTTGG
	Reverse Primer	CAGGGTAAGGGACAAATC
**B9J08_004797/HGT7**	Forward Primer	CGGTAACAACTACTTCTTCTAC
	Reverse Primer	AGAGTAGATGATGAAACACAC
**B9J08_000938/HGT10**	Forward Primer	CTCGGTCAGTTCGTTATC
	Reverse Primer	CCAATCTACCTCTGTTCTC
**B9J08_002250/HGT12**	Forward Primer	GGGTATCGGTATGTTCTTC
	Reverse Primer	CCTTGATATCGTCGTACTC
**B9J08_001015/HGT13**	Forward Primer	AGCTTGTGTTTCCTCTCTCTATG
	Reverse Primer	GACACCCTGATATCCTCAATCAA
**B9J08_004264/HGT14**	Forward Primer	GGAAGACGTATCTCGCTCATTATT
	Reverse Primer	TGCGATGATTCTTCCTCTTATGG
**B9J08_000582/HGT16**	Forward Primer	CACTTACTACTCTCCAACCATCTTC
	Reverse Primer	CGTATGCTCCAATGTACCACATA
**B9J08_002660/HGT17**	Forward Primer	GTTGCATCTCTGGTAGTG

	Reverse Primer	GAGGTGATGTTGGACTTC
**B9J08_000347/HGT18**	Forward Primer	GCGTTGAAGTGGAACAATGATAAC
	Reverse Primer	CCTCGTTTCTCACAAGGGAATAC
**B9J08_005025/HGT19**	Forward Primer	GGGAAAGGTAGACATGAG
	Reverse Primer	CTGGTAGGATGTTTCCTC

Supplementary Table S3: List of oligonucleotides used for the construction of the gene knockout

Gene ID/Name	Primers	5' - 3' Sequences
**B9J08_005568/ HGT2**	P1 Forward	GTTCAAGCTCTAGTCGATTT
	P5 Forward	AGGTGGGTTCTTTGCTATTT
	P6 Reverse	gcgtcgacctgcagcgtacgAGAGGATGAGAGAAGCAATCAG
	P13 Forward	CTACATCAGTTTCGGTTTGAG
	P14 Reverse	TAGAACCAGAATCAGGGTATG
	P7 Forward	cgacggtgtcggtctcgtagGGTTTGTAGTCGAGCATAAC
	P8 Reverse	GCCTCAATCCATACACATAC
	P4 Reverse	TTAAGTTTGGCGAGATCATT
**B9J08_002259/ HGT4**	P1 Forward	TCCAAGTTTCGTAGTAGTTTAG
	P5 Forward	GAGTTGAACGTTCTCCTTTATT
	P6 Reverse	gcgtcgacctgcagcgtacgGGGCTGAAATAGTATGCGAAATG
	P13 Forward	GTGGAATGAGCTTCTGTAATTT
	P14 Reverse	TGATAGGTGGTGGAAGTTGTTAG
	P7 Forward	cgacggtgtcggtctcgtagGAGCAGGATCTTGAGTCTTTCA
	P8 Reverse	CTCTGCTGATGTATCTTGAATA
	P4 Reverse	TTTCGGCTTTATGCTTATCC
**B9J08_002646/ HXT5**	P1 Forward	AGGGCTTTGTTGAGAGGAGCG
	P5 Forward	TGTGGGGAATGCATTTAGACG
	P6 Reverse	gcgtcgacctgcagcgtacgGAGGAATAATAAGATGCCCGTCGTGTC
	P13 Forward	GGTAACTCGTTAGTGGACCGC
	P14 Reverse	AATCTCCACCCGCCCTTAACAG
	P7 Forward	cgacggtgtcggtctcgtagACCTCTACCTGCTGTACCCC
	P8 Reverse	TTGAAGTATATCGGCAGTCTCC
	P4 Reverse	ATCATTAGGCTGTGCAGACTCCGAGC
**B9J08_002250/ HGT12**	P1 Forward	CGCCAGCCGTATATAACAATGC
	P5 Forward	CAGACCCCTCAGTGCTACGC

	P6 Reverse	gcgtcgacctgcagcgtacgAAGAGAAGGGTAAGATCCAGGGG
	P13 Forward	TTTGGCTGCTGTGTTCAACC
	P14 Reverse	GTAGGCAATTCCCCAGTTCC
	P7 Forward	cgacggtgtcggtctcgtagAAGAAGAGGCTTGCTATTTGATCC
	P8 Reverse	CTTCTTCCGATTCGCCTACAG
	P4 Reverse	CGCCAGCCGTATATAACAATGC
**B9J08_001015/ HGT13**	P1 Forward	GTAATGCTTGTTTACGCCAGTC
	P5 Forward	CAATTTCTTGGACAATGCGACTC
	P6 Reverse	gcgtcgacctgcagcgtacgGATGGAATGAGAGACAAGACTAC
	P13 Forward	GATCACATTCGGTATTTGTATTTC
	P14 Reverse	GATAAGTGAAGCCAAAAAGTAC
	P7 Forward	cgacggtgtcggtctcgtagATTCTCATATAATAATCGAAATTC
	P8 Reverse	GAATACTCGCCTAATGAAGAATG
	P4 Reverse	CTTAGCAGGAAATGAGAAGAG
**B9J08_005025/ HGT19**	P1 Forward	GATTACGTCATTTTGTCCTCC
	P5 Forward	GCTTGTTTCCAAAAATTGTGTCC
	P6 Reverse	gcgtcgacctgcagcgtacgTTGCAGCCACAGGTACAGGAAG
	P13 Forward	GACATGTTTCATTTCGGCCATC
	P14 Reverse	GCGAAAAGGAAATTGATCATAC
	P7 Forward	cgacggtgtcggtctcgtagGTAATGATTTAATTTTATAC
	P8 Reverse	GTAACTATTGTATTTGAAGGAC
	P4 Reverse	GTACTGCTGGTTAAGGACATG
